# Understanding the implementation and sustainability needs of evidence-based programs for racial and ethnic minoritized older adults in under-resourced communities with limited aging services

**DOI:** 10.1186/s12913-024-10925-0

**Published:** 2024-04-13

**Authors:** Yelba Castellon-Lopez, Savanna L. Carson, Katherine T. Ward, Karina D. Ramirez, Lynn Phan Vo, Tony Kuo, Teresa Seeman, Stefanie D. Vassar, Laura Trejo, Ellen Eidem, María P. Aranda, Arleen F. Brown

**Affiliations:** 1 Department of Biomedical Sciences, Cedars-Sinai Medical Center, Cancer Research Center for Health Equity, Samuel Oschin Comprehensive Cancer Institute, Los Angeles, CA USA; 2 Department of Medicine, Division of General Internal Medicine-Health Services Research, David Geffen School of Medicine, University of California, Los Angeles, CA USA; 3 Department of Medicine, Section of Geriatrics, LAC/Harbor-UCLA Medical Center, Torrance, CA USA; 4 Department of Medicine, Division of Geriatrics, David Geffen School of Medicine, University of California, Los Angeles, CA USA; 5 Department of Family Medicine, David Geffen School of Medicine, University of California, Los Angeles, CA USA; 6 Department of Epidemiology, UCLA Fielding School of Public Health, Los Angeles, CA USA; 7 Los Angeles County Department of Public Health, Los Angeles, CA USA; 8 Department of Geriatrics, David Geffen School of Medicine, University of California, Los Angeles, CA USA; 9 Los Angeles County Aging and Disabilities Department, Los Angeles, CA USA; 10 USC Edward R. Roybal Institute on Aging, USC Suzanne Dworak-Peck School of Social Work, Los Angeles, CA USA

**Keywords:** Community health services, Disease management, Aging, Evidence-based practice, Health equity, Older adults

## Abstract

**Background:**

Evidence-based programs (EBPs) for older adults effectively improve health outcomes. However, there is a limited understanding of the unique needs of service providers as they consider adopting, implementing, and maintaining programs for older minority adults in low-income communities with limited aging services.

**Methods:**

We conducted semi-structured interviews with key informants of community-based organizations (CBOs) to understand implementation and sustainability needs of CBOs within four racial and ethnically diverse Los Angeles County geographic areas. We performed thematic analysis of interview transcripts.

**Results:**

Interviews were conducted with representatives from 25 senior-serving agencies providing aging-related EBPs. CBO representatives reported implementing EBPs in 8 domains: Falls Prevention (68%), Mental Health (64%), Caregiver Health (48%), Chronic Disease Management (48%), Diabetes Management (36%), Arthritis Management (28%), Physical Activity (24%), and Multiple Conditions Management (8%). Themes are presented using the six domains of the Bass and Judge framework for factors impacting successful and sustained EBP implementation. CBOs in low-income and diverse communities described unique challenges with tailoring interventions based on local community context (literacy, language), cultural context, and locally available resources (technology, safe community spaces, transportation) and faced resource-intensive administrative burdens through staff turnover, data collection, sustainable funding, and networking.

**Conclusions:**

Serving racial and ethnic communities has unique challenges that require tailored approaches and additional resources to ensure equitable access to EBPs for all communities. We describe suggestions for enhancing the effective adoption of EBPs among service agencies in under-resourced and diverse aging communities serving populations with aging-related health disparities.

**Supplementary Information:**

The online version contains supplementary material available at 10.1186/s12913-024-10925-0.

## Contributions to the literature:

- The research conducted in Los Angeles County identified facilitators and barriers in implementing and sustaining evidence-based practices (EBPs) for community-based organizations (CBOs) serving low-income, diverse older adults.
- The study highlighted the importance of adapting EBPs to the specific needs of older adults in under-resourced settings including language preferences, engagement, and retention strategies, and addressing barriers related to technology and access to healthcare services.
- To promote health equity, institutions and policies should encourage cultural congruence, understanding, and partnerships with community organizations as crucial factors for effective implementation of EBPs, particularly in diverse and low-income communities.

## Background

Evidence-based programs (EBPs) offer proven ways to promote health and prevent disease among older adults based on research documenting health benefits [1]. EBPs, including chronic disease management, physical activity, and nutrition programs, have increased self-efficacy, decreased health service utilization, and enabled participants to adopt healthy self-management behaviors [2]. EBPs have become prominent in aging services due to policies and registration requiring health prevention and promotion activities to be evidence-based [3, 4]. Despite strong evidence that EBPs can improve health outcomes, our understanding of their use and effectiveness in diverse communities and under-resourced settings is limited. Understanding the unique challenges of implementing EBPs in diverse, low-income communities, defined as those with limited aging services and a large proportion of racial and ethnic minority aging residents with high social needs or disproportionate poor health outcomes, is especially essential to promoting aging health services equity [5]. Herein, we define diverse, low-income communities as areas with a majority (more than 50% non-white of population aged 65+) of racial and ethnic minority aging residents with high social needs (lower median incomes) or disproportionate poor health outcomes (disproportionate rates of chronic disease). While there has been a strong focus on the dissemination and implementation of EBPs at a national level, much remains to be learned about the delivery of aging programs at the local level, particularly among low-income and ethnic minority communities [6, 7] who face disparities in illness rates, self-rated health, and mortality [8–10].

One challenge identified in implementing EBPs across diverse communities is balancing cultural adaptations with maintaining fidelity [11]. Many EBPs were developed, implemented, and tested in well-resourced, well-known, university-supported settings with administrative infrastructure not found in many local, community-based organizations (CBOs) that offer EBPs and serve minoritized or low-income groups [12, 13]. While several programs focused on mental health, substance abuse, and children’s health have been successfully tailored and implemented in diverse settings, there are substantial gaps in understanding how EBPs for older adults can be implemented in racial and ethnic minoritized or under-resourced communities and the community-based organizations CBOs that administer said programs [14, 15]. Additionally, there is an inadequate understanding of the needs of community-based organizations (CBOs) or service providers in these communities as they consider adopting, implementing, and maintaining programs for older minoritized adults [16, 17].

This study was one of a series of projects selected to enhance healthy aging in Los Angeles County [18]. Older adults comprise over 20% of Los Angeles County’s population, a ratio expected to increase rapidly to nearly one in four Angelenos by 2030 [19, 20]. Racial and ethnically diverse older adults make up over 60% of the aging population in the county and have higher rates and inadequate control of chronic conditions and preventable disease [19, 20]. Community-based organizations are essential in delivering EBPs to diverse older adults living in under-resourced communities in Los Angeles County. This study aims to understand the perspectives of CBO stakeholders who have implemented EBPs for diverse older adults https://ncoa.org/evidence-based-programs in Los Angeles County living in low-income communities that serve primarily racial and ethnic minorities facing aging -related health disparities. We focus our findings using Bass and Judge’s six characteristics of EBPs which impact successful implementation to report our findings (i.e., Community Characteristics, Intra-Organizational Characteristics, Evidence-Based Program Characteristics, Fidelity, Staffing and Training, and Marketing, Cost, and Payment Sources) [15]. We selected this framework because it offers important implications for the role of education about EBPs through an organizational lens that can be translated into actionable recommendations for researchers [21]. In this study, we characterize the barriers and facilitators to implementing EBPs to promote healthy aging, chronic disease prevention, and management among aging, racial and ethnic minoritized populations in Los Angeles County through the perspective of CBOs and local providers who deliver the programs. Based on community partner feedback, we propose “real world” recommendations to overcome barriers to implementing EBPs while accounting for participants' racial and ethnic, cultural, and language diversity. We describe suggestions for EBP researchers, funders, and policymakers to enhance the adoption of EBPs among CBO service agencies in under-resourced and racially and ethnic minoritized communities.

## Methods

We conducted in-depth, in-person, semi-structured interviews from January 2015 to April 2015 with representatives of service agencies providing programming, training, or support for evidence-based aging programs within four racial and ethnically diverse Los Angeles County geographic areas. Four Los Angeles County Service Planning Areas (SPAs)--Metro, South Los Angeles, East Los Angeles, and South Bay--were identified as areas with low median incomes, disproportionate rates of chronic disease above 50 years of age (e.g., arthritis, diabetes, hypertension), a substantial minority aging population (over 50% non-white), and considered areas of high priority for aging services in the county [20]. Individual interviews were conducted to explore individual agency perspectives on implementing aging EBPs for health promotion, physical activity, and chronic disease management. This study was part of a larger evaluation project in collaboration with the county. The interview guide was developed in conjunction with community providers and asked explicitly about EBPs listed on the National Council on Aging’s list of Approved Evidence-Based Health Promotion/Disease Prevention Programs (Title IIID of the Older Americans Act) [1]. The full interview guide can be found in Appendix 1. Sample qualitative questions are listed in Table 1.

**Table 1:** Sample qualitative questions

**Select Interview Questions**^**a**^
• What evidence-based programs/interventions is your organization currently implementing? (Included list selection of common aging EBPs)
• How does your organization come to identify these interventions as evidenced-based?
• What populations (or groups) is this EBP specifically targeting or intended for?
• How do you typically evaluate your EBPs? If you could have any information that would help you determine how effective a program is, what information would that be? How would this information be helpful to you? Why is this information important to your organization?
• Did your organization modify, adapt, or specifically tailor any of the EBPs from the original format? If so, in what ways were programs modified?
• Describe typical organizational costs related to the implementation of EBPs for older adults.
• What factors have ultimately influenced or facilitated your organization’s decision or ability to deliver EBPs for older adults?
• Describe the challenges or barriers that your agency/organization has faced in implementing EBPs for older adults
• Are there unique issues to consider in implementing EBPs for older adults compared to EBPs for adults in general (e.g., any unique challenges/barriers, costs, opportunities/facilitators)?
• Are there unique issues to consider in the implementation of EBPs for minority older adults in comparison to non-minority older adults (e.g., any unique challenges/barriers, costs, opportunities/facilitators)?
• What EBPs were offered in the past five years but are now discontinued?
• Why were they discontinued?
• What would adopting an additional EBP for older adults in your agency/organization take?

^a^Full guide available upon request

### Study sample

Organizations in the four target SPA areas were identified through agencies known by the Los Angeles County Aging and Disabilities Department, the City of Los Angeles, and Los Angeles County Area Agencies on Aging (AAA). As no complete list of EBP-providing CBOs was available, during the interviews, other organizations were identified through snowball sampling by asking interviewees about other known community organizations offering evidence-based aging programs. Agency recruitment ended when no new agency contacts were provided in the SPA areas of interest. Informed consent was obtained from all individual participants included in the study. This study was reviewed and approved by the Institutional Review Board (IRB#14-001703).

### Data collection

We conducted a pre-interview survey on interviewee demographics and agency characteristics and a semi-structured interview with participants on experiences with EBP implementation, delivery, and sustainability. Interview questions topics related to perspectives on the type and location of EBPs provided; how the organization defined and learned about EBPs, populations served, whether and how agencies modified, adapted, or specifically tailored EBPs; unique issues to consider in the implementation of EBPs for older adults and minority older adults; and organizational support for, barriers to, evaluation of, and fidelity to EBP implementation and sustainability (See Table 1, for sample questions from the interview guide). Three pilot interviews were conducted with aging stakeholders to gain feedback on refining and modifying the interview questions for CBOs, specifically, two county officials and one aging researcher. These stakeholder pilot interviews were retained in the analyses as they provided relevant policy perspectives in their interactions with CBOs implementing EBPs. Questions were sent to interviewees beforehand to prepare for the interview and to gather details on the types and ranges of EBP programs or services offered. Interviews were held on-site at the local agency in a place (e.g., an office or conference room) chosen by the participant, and interviews lasted between 45 and 90 minutes. Interviews were recorded and transcribed. Interview notes and surveys pre-filled by the agencies were scanned and reviewed with the transcripts for accuracy and verification.

After preliminary data were compiled, we completed a modified Delphi approach with interview participants and other local AAA stakeholders to validate, refine, and confirm themes from the interviews and establish an initial list of priorities, challenges, barriers, and implementation strategies for EBPs in diverse communities in LA County. A teleconference was held to disseminate the findings by reviewing the results of both the interviews and the panel.

### Data analysis

Qualitative analysis was performed on interview transcripts using a critical realist and reflexive six-phase thematic analysis approach [22–24] to consider social, cultural, economic, structural, and political factors within EBP implementation [25]. The analysis consisted of one primary coder (SC) reading the transcripts repeatedly and then iterative coding, noting reflections in memos using Atlas.Ti. The coder did not conduct the interviews nor have insight into non-verbal cues or responses from participants that may have occurred beyond the transcripts, field notes, and Delphi-panel meeting notes. Thus, the research team provided feedback on the provisional reflections, coding scheme, coding definitions, and themes and recommended revisions or clarification throughout the analysis. Final themes were generated, revised, and defined based on reviewing provisional themes, verifying patterns across the data set, and checking how well candidate themes reflected the data, field notes, and supplemental meeting notes.

We organized themes that emerged into six domains identified by Bass and Judge as important characteristics in the successful implementation of EBPs through the lens of the key informants. The themes identified are organized using the following domains: (1) Community characteristics, (2) Intra -organizational characteristics, (3) evidence-based program characteristics, (4) fidelity, (5) staffing and training, and (6) marketing, cost, and payment sources [15]. We further described challenges relevant to (a) the general aging population and those specific to (b) racial and ethnic minoritized older adults in under-resourced community settings.

## Results

We conducted 25 in-depth, in-person, semi-structured interviews throughout Los Angeles County focus areas (SPAs 4, 6, 7, 8). Characteristics of these CBOs are reported in Table 2. The CBOs implemented EBPs in 8 domains: Falls Prevention (68%), Mental Health (64%), Caregiver Health (48%), Chronic Disease Management (48%), Diabetes Management (36%), Arthritis Management (28%), Physical Activity (24%), and Multiple Conditions Management (8%), described in Table 2.

**Table 2:** Characteristics of community-based organizations

**Organization Characteristics (*n*=25)**	
Type of Organization (select all that apply)	N (%)
Community-Based Organization	10
Multipurpose Social Service Organization or Senior Center	6
Housing / Residential (e.g., senior housing, retirement community)	4
Health Care Organization	4
Recreational Organization	2
Government State / County / Municipal	2
Area Agency on Aging	1
Number of Employees	
<25	4 (16)
25 to 50	2 (8)
51 to 99	3 (12)
100 to 200	4 (16)
>200	10 (40)
Missing	2 (8)
Los Angeles County Service Provider Area (SPA) Served *(check all that apply)*	
4-Metro	9 (36)
6-South Los Angeles	6 (24)
7-East Los Angeles	7 (28)
8-South Bay	5 (25)
County-wide agency	4 (16)
Annual Budget (US Dollars)	
<$1 million	3 (12)
$1 million to $9 million	6 (24)
$10 million to $100 million	7 (28)
>$100 million	3 (12)
Missing	6 (24)
Financial Support for Agencies	
General Funds	5 (20)
Federal, state, or county grant programs	15 (60)
Time-limited grants	3 (12)
Insurance reimbursement	7 (28)
Foundation or Corporate funding	9 (36)
Donations	11 (44)
Participant payments	11 (44)
Other	7 (28)
Financial Support for EBPs*	
Payers (e.g., Medi-Cal or Private Insurance)	4 (16)
Participant Payments	5 (25)
Federal, state, or county grant programs	10 (40)
Foundation or Corporate funding	11 (44)
General Funds	3 (8)
Donations	1 (8)
Aging-related EBPs Implemented by CBOs	
Falls Prevention	17 (68)
Caregiver Health	12 (48)
Chronic Disease Management	12 (48)
Diabetes Management	9 (36)
Arthritis Management	7 (28)
Physical Activity	6 (24)
Multiple Chronic Conditions	2 (8)

While many CBO participants described the value of EBPs, for example, “*It’s not so much teaching them as giving them techniques to do it for themselves*” (CBO #6), we focus our results on the challenges in implementing and sustaining EBPs for Los Angeles County CBOs serving low-income, diverse adults. Table 3 describes barriers to implementation themes below.

**Table 3:** Barriers to implementation of aging-related EBPs in racial and ethnically minoritized serving organizations in under-resourced settings

**EBP Characteristics** [15]	**General Aging Populations**	** Racial and Ethnic Minoritized Groups and Under-resourced Setting Considerations**
**Community characteristics**	• Limited range of accessibility: hearing, seeing, mobility, cognitive impairment • Varying levels of independence • Emotional barriers related to lack of social support, loss of loved ones • Difficulty with paperwork (privacy concerns and/or finding it hard to read) • Social isolation and anxiety about going somewhere new if not aware of the EBP-providing organization • Need for flexibility in participation requirements (i.e., doctors appointments, health, wellbeing)	• Range of literacy levels • Language preference (including language heterogeneity) not depicted in EBP • Competing responsibilities: home, caretaking, job responsibilities, basic needs, religion, etc. • Affordability and accessibility of technology (phones, internet) affecting awareness or participation • Access to safe, reliable transportation or lack of walkability in the community • Low awareness of EBPs and prevention programs in the community • Lack of aging related services in the area
**Intra organizations characteristics**	• The administrative burden of program management • Variable leadership support and buy-in for implementing EBPs • The administrative burden of data collection and submission for EBPs, including multiple required submissions and copies for EBP developers, funders, etc. • Lack of ability and capacity building to do internal program improvement and data collection • Opportunity to collaborate with other community agencies to distribute work and promote program sustainability	• Opportunity, need, and administrative time burden for building programs and EBP partnerships within trusted institutions (faith-based programs, programs within public housing) • Leadership not understanding population needs, assets, values, culture(s)
**Evidence-based program characteristics**	• Need for data transparency from EBP developers or funders for feedback for CBO quality improvement Data restrictions (ability to add questions, submission requirements, etc.) • Intrusive data gathering on participants • Challenge of senior engagement in class length and time of day for classes (takes time to find the “right” timing for the population served) • Eligibility is limiting to serving the community’s needs	• Lacking cultural relevance in language or materials and diverse representation of examples in the curriculum • Tailor curriculum to lower literacy levels to increase accessibility • Should address social determinants (food insecurity, poverty, etc.) • Language heterogeneity beyond English or Spanish
**Fidelity**	• Need tools, training, or resources to ensure fidelity	• The tension between the need for cultural adaptations and maintaining fidelity • Need for flexibility in attendance for elderly and high SES-burdened population • There is a need to build trust in local organizations to establish programs which cater to the local population
**Staffing and Training**	• The administrative burden of staff management: staff or volunteer turnover, the hidden costs of volunteers, workforce development • Training takes time and has a cost • Capacity training to recruit, train, and deliver the program	• Lack of volunteer staff or pool of volunteers that looks like the population served • Lack of program evaluation skills and perceived importance of collecting meaningful data • Staff qualifications (limited professional, educational, or training experience)
**Marketing, cost, payment sources**	• Outreach and recruitment take time, and staff not typically covered • Needs for referral sources • Cost of training can be expensive and takes time • Financial burden of EBP implementation: evaluation, training time and cost, teaching, fidelity	• Need consistent funding streams to promote uninterrupted program delivery • Diverse representation in marketing materials • Offer incentives

1. Community Characteristics that should be considered when implementing EBPs with older adults refer to individual and community-level characteristics, including social norms and values that may influence community preferences and older adult behaviors that are typically outside the organization’s control [15].
  - *General older adult population*: Our study participants reported the importance of adaptations targeted to the needs of older adults when developing and implementing EBPs that serve this community. Important adaptations to consider in older adults include addressing the needs of those with visual, hearing, or cognitive impairment; participant level of independence; providing the opportunity to have flexibility in participation requirements; and consideration for the emotional needs of older adults, such as losing close friends and different levels of support systems. Participants further pointed out that interventions need to consider the mental health needs of aging populations due to the availability of issues they often face concurrently, such as social isolation, loss of loved ones, or lack of social support. These adaptations did not exist for many EBP programs implemented by the participating CBOs. As one CBO representative noted, *“if, for example, [the intervention is] offering something where they use a visual, sometimes our clients have macular degeneration or vision issues, so … that is a challenge”* (CBO #17).
  - *Racial and ethnic minoritized community serving organizations in under-resourced settings*: Key stakeholders in organizations serving low-income and diverse populations described specific community characteristics that present challenges with implementing EBPs, including the need to increase community awareness of existing EBPs, the range of population literacy levels, varying language preferences, and challenges with engagement and retention due to competing demands such as work or caretaking responsibilities. Older adults in low-income communities may also have limited resources, struggling with the affordability and accessibility of technology, such as mobile phones and internet access. One agency pointed out that “*what ends up happening is they have prepaid phones, or they don’t have enough credit to open a phone line, so they’re on a prepaid phone card…we might call them, and they ran out of minutes, or they switched phones already because they can’t afford an expensive phone”* (CBO #19) making it difficult to follow-up consistently with participants. Stakeholders shared community characteristics that may create challenges for organizations serving low-income ethnic and racially diverse older adult EBP participants, including limited access to stable housing or transient populations, food deserts limiting access to healthy food options, park proximity, and sub-optimal neighborhood walkability. As noted by one agency leader, “*the reality is that some [older adults], again for safety reasons, or because of limited mobility or transportation, or they just don’t really want to come out of their building, they don’t come to the centers*” (CBO #16).
2. Intra-organization characteristics refer to organization-level barriers to delivering an EBP. Factors that make for the successful delivery of EBPs are leadership and staff buy-in, the relationship of a new EBP program with other existing programs within the organization, and the adoption of EBPs by organizational staff members.
  - *General older adult population:* A majority of CBOs implementing EBPs in our study reported that doing so requires organizational capacity building of infrastructure for data collection, program delivery, data entry, and ongoing internal program management, all of which lead to additional administrative burdens on staff. CBO participants explained how evaluations and surveys for EBPs add administrative duties, stating “*there is so much copying [of evaluations]”* (CBO #11) and providing multiple reports to funders or academic program developers*.* Participants suggested providing administrative support and training to help manage CBO volunteers and staff facilitating EBPs, explaining, “*volunteers aren’t free. We got to screen them; somebody has to supervise them. They don’t show up, and you’ve got to backfill, and there’s no real honest conversation about the infrastructure it takes*” (CBO #11).
  - *Racial and ethnic minoritized community serving organizations in under-resourced settings*: An important issue related to leadership buy-in raised by organizations serving diverse and low-income communities is the lack of cultural congruence and understanding between organizational leadership and the communities they serve. One organization noted, “*I think that’s a challenge from an organizational perspective to be sure that our leaders are representative of the cultures that we are serving, and also that [leadership] folks have a good understanding of key cultural concepts of those different cultures we’re serving”* (CBO #15). Similarly, minority-serving organizations report that EBPs work best when there is congruence in the racial and ethnic makeup between the staff that implements the program and the population they serve. Lastly, participants reported that given the history of mistrust and marginalization of minoritized communities, EBPs may not always recognize or value the importance of building community partnerships, such as with faith-based organizations or other social service agencies, to further gain community trust and expand outreach. This is especially helpful with recruitment and retention.
3. Evidence-based program characteristics are important factors CBOs should consider when adopting EBP programs. These include (1) compatibility with the mission and goals of the parent organization, (2) strength of the research findings, (3) subject recruitment experiences in the original research, and (4) attrition rates before study completion [15]. Finding an EBP aligning with the host organization’s mission allows for improved capitalization of existing organizational resources such as staff competencies, established reputations, and community alliances [15]. It is also important to evaluate the strengths of the research and consider the consistency and significance of the benefits for different populations, given that anticipated outcome benefits of the original research may not retain their meaning or clinical significance across diverse populations.
  - *General older adult population:* CBO representatives in our study identified specific challenges related to recruiting older adults, including lengthy data collection instruments and the time allotted to program sessions for the older adult population. For example, several organization representatives mentioned that “*[older adults do not like filling out all the paperwork*” (CBO #18) and that older adults may perceive data collection efforts as intrusive because agencies request what they perceive to be personal information. In addition, some agencies noted resistance from partnering sites in recruitment efforts, citing, “*no, there is no way you can have my [older adults] sitting in your class for two hours*” (CBO #16). Organizations recommended increased transparency about EBP outcomes, including allowing CBOs to view their outcomes data for internal quality improvement efforts. As one CBO stakeholder stated, “*wouldn’t it be cool if they could give us feedback? Hey, in the last year, you did “x” number of groups, and here [is] the feedback on your groups because we are sending all of that stuff to them, but we don’t have any [feedback]. For all I know, they hate one of the facilitators, and nobody has told me*” (CBO #7).
  - *Racial and ethnic minoritized community serving organizations in under-resourced settings:* Additional considerations raised by organizations serving low-income and diverse older adults included cultural representation in program outreach and recruitment materials and the development of culturally sensitive curricula. Many organizations noted the need for language congruence, culturally specific curriculum translations, and addressing social determinants of health and barriers to participation in under-resourced community setting. One participating CBO noted that EBPs, “*have been tested out on a very mainstream middle America population… a lot of things don’t translate down into the community, whether it’s cultural beliefs or stigma or even language with the material. It just doesn’t always work”* (CBO #21). Moreover, agencies identified the need for improved accessibility to programmatic content through adaptations for low-literacy populations, stating, “*Not everyone is reading at a 12*^*th*^*-grade reading level, so how do we make things more accessible”* (CBO #21).
4. Fidelity refers to the original delivery and design of the EBP. Fidelity promotes EBP effectiveness and requires CBOs and service providers to follow the proposed curriculum with minimal variation. Implementation in community settings requires explicit directions, materials, manuals, record-keeping, and monitoring to ensure fidelity [15].
  - *General older adult population:* Participants in our study described a need for ongoing support in managing fidelity in “real-world” settings. Participants asked for additional tools, continuing training, or resources for routine fidelity management, mainly due to the variable skills of volunteer or paid facilitators who are new to EBP delivery. Participants also described the unforeseen administrative and management burden of ensuring fidelity. One participant described how additional factors could influence fidelity, such as multiple training sites and numerous facilitators, explaining, “*some volunteers, although they go into the training, they may not follow the guidelines, they may not stick with the manual. Because they’re on their own out in the community, unless I go out and supervise them and then I can’t do that every single time, so how to teach them about the fidelity, how to maintain the fidelity and how to supervise them, that’s a challenge*” (CBO #8).
  - *Racial and ethnic minoritized community serving organizations in under-resourced settings*: CBO stakeholders described additional challenges with maintaining fidelity when working with racial and ethnically diverse older adults in under-resourced settings, particularly when considering how to maintain audience understanding, the need for cultural adaptations, and how to provide the most community-relevant programming to residents. Participants describe a tension between the importance of fidelity and the need for cultural adaptation, mainly depending on the population served. One participant explains how one EBP program is “*so difficult to apply to every single ethnic group because we’re all different; we’re all very diverse. …the difficult part is how to create a program that is specifically good for Cantonese, African American, [and] Japanese American [populations]. I think that’s important, but it’s very difficult if we want to maintain fidelity*” (CBO #8). Often, funder-required outcomes require ensuring fidelity through pre-specified participation and attendance levels, which may present challenges in highly burdened, low-SES communities. As one interviewee further explains, “*A good percentage is not going to complete [the EBP program]… older adults don’t tend to want to be pinned down unless there’s some real incentive... and my experience has been, you know, I’ve seen statistics other people have published and things (about attendance), but I know ultimately the reality …every day I’m in the trenches*” (CBO #20). Many local organizations described the necessary adaption of programs for cultural relevancy, population-specific considerations, or language translation (see theme 3), but restrictions by funders or EBP developers on adaptations by local CBOs may limit EBP effectiveness in certain communities. Some participants perceived these restrictions as a lack of trust in CBO delivery of the EBP, “*It’s great to have the evidence of research around it, but at some point, you have to trust the community to really use their brains and their local resources to implement these types of programs because otherwise they won’t be used, and the community’s not going to benefit*” (CBO #15).
5. Staffing and training refer to the need for hiring, training, staffing, supporting, and supervising, from implementation to sustainability of EBPs.
  - *General older adult population:* Participants from CBOs described the high administrative burden of managing staff, adhering to training requirements, supervision, and workforce development, often citing the difficulty of volunteer management, staff turnover, and updating training. These challenges also influenced program sustainability.
  - *Racial and ethnic minoritized community serving organizations in under-resourced settings*: Among CBOs in under-resourced settings serving minoritized populations, respondents described limited capacity to hire, maintain, train, fund, and supervise qualified staff within a resource-limited community organization, especially those representing the local population served. A participant described a need for additional structural oversight for staffing EBPs, saying, “*The challenge is really figuring out what’s that structure and, again, because I don’t think it can just be done with volunteers. There must be some structural umbrella and support over it*” (CBO #7). Another participant describes the difficulty in finding staff or volunteers within the community to meet the EBP requirements, stating, “*As we move towards insurance reimbursement, I feel like the* [university name redacted] *folks are locking down on fidelity. And now you’ve made it so complicated that I can’t have lay leaders in charge of this. So, okay, now I have to hire somebody to run the program you’re going to reimburse me for, which just added cost to my system that may not equal what you’re reimbursing me for. And that’s the spot where we get trapped. And then, I can’t really then have lay leaders*” (CBO #5). Many CBO leaders emphasized the importance of the “right” staff who are representative of the local population to provide culturally and geographically congruent instruction. As a participant explains, “*culture is the big one. That’s the tough one. It’s more than just translating. You’ve got to have the right staff*” (CBO #2).
6. Marketing, cost, and payment sources refer to the financing-related issues and business plans for implementing and managing EBPs to promote sustainability.
  - *General older adult population:* CBO representatives in our study described financial barriers to implementing EBPs for older adults, including funding, so that did not include compensation for time and financial investment in community outreach and recruitment, the high cost of training staff, and budgets that are affected by staff turnover or availability, and the high cost of implementing a new EBP with evaluation, teaching, training, and fidelity requirements. As one participant explained, “*whether you’re just going out and walking the streets for people or whether you’re going door to door or you’re calling and mailing, it’s expensive to recruit participants*” (CBO #2). CBOs also described the importance and lack of a strong referral network and the need to increase awareness of EBPs by health and social systems. As one participant explained, it was important for physicians to believe *“in programs like this and not just refer somebody but really understand how this program fits into the continuum of care… and how with the whole transformation of health care now, how programs like these are going to be vital*” (CBO #25).
  - *Racial and ethnic minoritized community serving organizations in under-resourced settings* In particular, representatives of CBOs serving racially and ethnically diverse populations or those with low-income described the need for consistent funding streams to promote uninterrupted program delivery, the need for diverse representation in marketing materials, and the desire to offer incentives to encourage participation in populations who have financial limitations. They emphasized the need to recognize that many minoritized older adults may experience mistrust in EBP participation due to the legacy of racism in research. Structural racism may play an important role, as participants may reside in communities with few available social resources, be unaware of those resources, or have other socioeconomic, logistical, or accessibility limitations for participating in EBPs. These factors should be considered by funders who provide EBPs in under-resourced communities serving diverse adults. With regard to the need for consistent funding, one participant raised concerns about the requirements community organizations would need to meet for medical reimbursement, explaining, “*while reimbursement is great and interest from health insurance companies is great, are we becoming so rigid and so medicalized that we’re really cutting these small, community-based organizations out of being able to offer it and reducing how many classes we can offer?*” (CBO #9).

Participants also described difficulty recruiting participants from communities with the most need for EBPs. For example, they decried the lack of representation in EBP outreach materials, stating that “*all of the material, you can see, the pictures, are very not diverse*” (CBO #13). They encouraged engaging promotional materials incorporating images and cultural factors representing the community of interest. CBO stakeholders also emphasized the need for equitable incentives for facilitating participation from communities with the most need for EBPs by recognizing the financial and non-financial costs of participation, stating, “*we really want to get the people that don’t have those resources that aren’t seeking help, the ones that are quiet, the ones that are not coming in… it’s hard to reach that population, and it’s a public health issue”* (CBO #19). Participants explained that incentives could include food, transportation, caregiver respite, and reimbursement for lost wages, as many older adults in lower SES communities remain employed because they have limited or are not eligible for retirement benefits.

## Discussion

Our findings describe barriers and facilitators to EBP implementation facing local CBOs when working with general older adults and diverse and low-income older adults with unique needs and challenges. We use the Bass and Judge framework to categorize perceived challenges encountered by CBOs. This is one of only a few studies examining challenges in sustaining EBP implementation from the perspective of CBOs who predominantly serve racial and ethnic minoritized older adults in under-resourced settings . CBOs in under-resourced communities focused on serving diverse older adults describe the strains involved in the implementation of EBPs for predominantly racial and ethnicy minoritized older adults, including the range of literacy levels, language preferences, and the need to account for disparities in access to resources such as limited technology, access to safe community spaces, and lack of transportation. CBOs faced the usual barriers of high staff turnover at the organization level, administrative burden on staff associated with data collection, and the need for collaboration with other community agencies to promote program sustainability. CBOs felt that EBPs often lacked cultural relevance and diverse representation in their curriculum. They also expressed the tension between maintaining fidelity and the need to build in flexibility for cultural adaptations. This tension is identified in other literature as the “fidelity-adaptation dilemma” which refers to the tradeoff between maintaining EBP fidelity and considering context appropriateness when implementing EBPs [26]. CBOs expressed a need for sustained funding streams, recognition, and investment in the additional effort required to engage racial and ethnic minoritized older adults in under-resourced settings. The conditions, modifications, and sustainability considerations for equitably implementing EBPs for racial and ethnically diverse older adults in under-resourced communities were significant yet under-recognized by those who developed and funded EBPs.

Previous studies have described several challenges experienced by CBOs in implementing EBPs among older adults, including the ability for the program to be adapted, [7, 27] ongoing training to ensure fidelity, [15] lack of trained staff and high staff turnover, [28] indirect costs associated with the implementation, management, and sustainability of EBPs in a variety of community settings (e.g., initial licensing and ongoing licensing), funding sources, staffing costs, local infrastructure, facilities available, and organization type [15]. This study adds to the existing literature by describing the unique challenges navigated by CBOs who serve racial and ethnic minoritized older adults who also live in under-resourced communities and are traditionally underrepresented in research and EBP trials [28–32]. Key informants also highlighted facilitators for implementing EBPs for CBOs serving racial and ethnic minoritized communities such as cultural representation among CBO leaders and staff with the communities they serve, established trust between the organization and community leading more community engagement, and the ability to provide language responsive, and place-based services. This study provides translational and implementation feedback and calls for future studies incorporating cultural tailoring, modification, and translational flexibility.

Our results also have policy implications. Based on feedback from agencies with experience translating EBPs for diverse minoritized older adults in under-resourced settings, we propose the following recommendations for researchers, funders, and policymakers summarized in Table 4. Recommendations promote ways in which EBP translation efforts should consider necessary adaptations or provide guidelines that allow for tailoring to accommodate the delivery of culturally congruent, linguistically relevant, and socioeconomically sensitive material that reflects a more racial and ethnically diverse demographic beyond the population in which the original curriculum was tested and evaluated. To promote health equity and cultural inclusivity, EBP developers should provide culturally diverse curricula and content sensitive to the social needs of and the underlying social determinants of health factors (access to healthy food/fresh vegetable sources, prepaid phones, reliance on public transportation, access to safe spaces to exercise, internet access, etc.) that can impact participants’ use of and retention in these programs. Additionally, the financial viability of EBPs will depend on the availability of third-party reimbursement (i.e., public or private health coverage), as grants and customer funding lack sustainability. In addition to these financial concerns, local CBOs need logistical support and strategic resources to sustain EBPs for vulnerable older adults.

**Table 4:** Key Informant recommendations for researchers, funders, and policymakers to implement evidence based practices in minoritized communities

**Community Characteristics**
• Design Interventions with a focus on equity and resource-limited settings. Develop customizable program options that minimize infrastructure demands, focusing on solutions that utilize available resources.
• Cultural and Local Adaptations are Key. Prioritize the inclusion of culturally relevant materials and training modules and offer intervention elements that can readilty be culturally tailored. Ensure the intervention reflects and respects the community it serves by incorporating community feedback early and throughout the development and implementation process.
• Champion Accessibility for All. Provide curriculum and material in multiple formats, languages, and tailored to diverse literacy levels. Allow for program flexibility. Incentivize Equitabily to Enhance Participation. Recognize that low-income and diverse populations may face more structural barriers to participation (i.e., gift cards, food, insurance-based incentives, etc.).
**Intra-Organizational**
• Build and Leverage Existing Systems for Cross-Agency Referrals for EBP promotion and awareness (e.g., health systems, medical providers, social service organizations serving aging populations, etc.)
• Strengthen Feedback Channels with a Commitment to Continuous Care: Establish consisten and transparent mechanism with referring providers to enhance program recognition and ensure continuous care (e.g., patient experiences, pre/post-EBP data, etc.).
**Evidence-based Program**
• Adapt EBP Curriculum for Diverse Populations. Provide guidelines for how curriculum may be modified for local populations (language, literacy level, scenarios, culturally tailored meals, etc.)
• Reflect Diversity in EBP and Outreach Efforts. incorporate diverse representation and people of color in EBP and outreach materials.
• Acknowledge and Address Social Determinants for Program Adherence. Tailor content to be sensitive to social determinants and support resources needed for program adherence (access to healthy food sources, prepaid phones, reliance on public transportation, access to safe spaces to exercise, internet access, etc.)
• Provide Culturally Relevant Translation and Tailoring.
• Provide technical assistance with data collection and submission to reduce local data administration.
• Share EBP Particiapnt Data for Local Enhancement and Quality Improvement.
**Fidelity**
• Provide guidance on adapting or local modification of EBPs to improve accessibility (limited literacy, reading proficiency, language translation, transportation, virtual sessions) while still valid to the original EBP.
• Conduct studies of equivalence for adapted, modified, or ability-to-customize EBPs so that concerns about fidelity do not undermine the use of effective programs.
**Staffing and training**
• Promote career advancement for local EBP staffing or volunteers such as EBP training certificates, e.g., “master training” or “certificate in health education.”
• Expand Medicare reimbursement model to more EBP programs.
**Marketing, cost, and payment sources**
• Develop sustainable funding or reimbursement streams to support implementation ramp-up and sustainability of EBP programming (i.e., staff turnover, volunteer management, participant recruitment efforts, evaluation, etc.)
• Provide customizable recruitment materials for EBP programs and marketing

There are several limitations to be considered in our study. This study was a convenience sample that only included Los Angeles County areas with limited aging -related EBP programs in low-income and racially and ethnically diverse settings. We did not include organizations serving other regions in Los Angeles County (specifically SPAs 1-3 and 5). However, as described in the results, some participating organizations compared differences in EBP implementation across their catchment areas in under-resourced and well-resourced regions. It is also noted that general challenges to implementing these programs are difficult to overcome regardless of the available resources. Our data was also collected in 2015. However, many of the barriers mentioned are still relevant today and are applicable across time. We examined the implementation of EBPs in general, filling a gap in existing literature. Further investigation is needed to understand best practices for implementing each specific EBP category in diverse settings, including under-resourced settings and racial and ethnic minoritized groups.

## Conclusion

Our study found that CBOs working with racial and ethnic minoritized older adults in under-resourced communities face additional challenges that should be considered by researchers, funders, and policymakers.. Providing high-quality assistance with translation and cultural tailoring are potential strategies that may reduce such barriers. In addition, funding mechanisms that support implementation and sustainability should provide additional support to account for higher social needs in these settings, including more staff turnover and higher cost and intensity of recruitment in older adults with multiple competing clinical and social demands. Lastly, long-term funding and reimbursement structures that are sensitive to the needs of community partners and CBOs should be considered if widespread dissemination is desired.

### Supplementary Information

**Supplementary Material 1.**

## Data Availability

The datasets used and/or analyzed during the current study are available from the corresponding author on reasonable request.

## Abbreviations

EBPs: Evidence-based programs
CBOs: Community-based Organizations
SPAs: Service Planning Areas
AAA: Area Agencies on Aging
LAC WDACS: Los Angeles County Department of Workforce Development, Aging and Community Services
SES: Social economic status
