# Supplementary material for: Understanding the implementation and sustainability needs of evidence-based programs for racial and ethnic minoritized older adults in under-resourced communities with limited aging services
Source: BMC Health Serv Res. 2024 Apr 13;24:466. doi: 10.1186/s12913-024-10925-0 (PMC11015605; doi:10.1186/s12913-024-10925-0)
Supplement: Supplementary file 1 — Supplementary Material 1. [file 12913_2024_10925_MOESM1_ESM.docx]

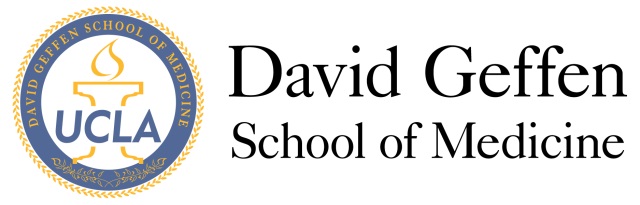

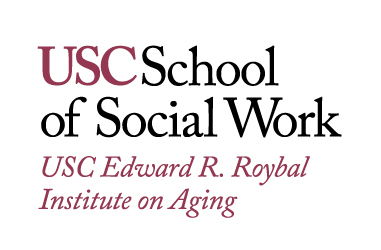


The Use of Evidence-Based Aging Initiatives in L.A. County and Developing Community-Informed Metrics for their Effectiveness

Bringing Evidence-Based Programs to Underrepresented Older Minorities

**TO THE INTERVIEWER:**

1. Prior to the interview, do the following:
   - Ask informant to have EBI data available, if possible: Q9-Q17
   - Be sure participant has read and understood the IRB Information Sheet.
   - Confirm whether or not the participant gives permission for audio recording.
   - Collect pertinent contact information; complete the Key Informant Contact Form.
2. Regular font: read to the participant (e.g., instructions, questions, response options).
3. *Italic font*: read to the participant. Also, display the appropriate SHOW CARD.
4. **Bold font**: do not read aloud. This includes directions to the interviewer about what to do next and marking of answers.
5. Do not read the response numbers associated with response options.
6. Choose the appropriate pronoun from phrases like “he/she” or “him/her.”

**PROCEED WITH INTERVIEW:**

Thank you once again for agreeing to participate in this study. It will help to gain a better understanding of what Evidence-Based Interventions (EBIs) are currently implemented for older adults in Los Angeles County. For this study, we define older adults as persons age 50 and over. Your participation in this study will help to ensure that we have the most comprehensive information possible.

There are four sections to this interview. In the first section we will ask information about your organization and the EBIs that your organization currently implements. In the second section, we will ask questions about the reach and utilization for each of the EBIs. In the third section of the interview, we will ask about evaluation, and the challenges and facilitators in implementing EBIs. Finally, in the fourth section of the interview, we will ask you about EBIs that may have been discontinued and about EBIs that you would like to implement in the future. It should take approximately 60 to 90 minutes.

**SECTION 1: ABOUT THE ORGANIZATION.**

First, let’s begin with questions about your organization/agency.

Q1 Which of the following describes your organization? You can choose more than one category. **Display the SHOW CARD, and check all that apply.**

|  | *Area Agency on Aging* |
| --- | --- |
|  | *State Department of Mental Health, Public Health, or Health Services* |
|  | *County Department of Mental Health, Public Health, or Health Services* |
|  | *Health Care Organization (hospital, clinic, treatment facility, etc.)* |
|  | *Faith-Based Organization (i.e., church, temple, etc.)* |
|  | *Recreational Organization (i.e., YMCA)* |
|  | *Municipal Government* |
|  | *Senior Center* |
|  | *Workplace* |
|  | *Multipurpose Social Service Organization* |
|  | *University-Affiliated Community Service Organization* |
|  | *Housing (e.g. senior housing or retirement community)* |
|  | *Residential Facility (e.g., assisted living facility or nursing home)* |
|  | *Other –specify:* |

Q2 About how many people does your organization employ?

|  | Fewer than 25 |
| --- | --- |
|  | 25 to 50 |
|  | 51 to 99 |
|  | 100 to 200 |
|  | More than 200 |

Q3 In what range is your organization’s annual budget?

|  | Less than $1 million |
| --- | --- |
|  | $1 million to $9 million |
|  | $10 million to $100 million |
|  | Over $100 million |

Q4 Where do your organization’s resources and funding come from? You can indicate more than one category. **Display the SHOW CARD, and check all that apply.**

|  | *On-going revenue stream (e.g., part of a regular line item in agency’s budget)* |
| --- | --- |
|  | *County, state, or federal grant programs* |
|  | *One or more time-limited grants* |
|  | *Insurance reimbursement (Medicare, Medi-Cal (Medicaid), HMO reimbursement, private insurance)* |
|  | *Foundation or corporation funding* |
|  | *Donations* |
|  | *Participant payments (fee-for-service, co-payments, private pay, etc.)* |
|  | *Other – specify:* |

Q5 Does your organization currently implement any Evidence-Based Interventions for older adults, defined as age 50 and over? YES NO **(If no, skip to Q18).**

Q6 What evidence-based programs/interventions is your organization currently implementing?

- **List each of the EBIs in the order that the Key Informant mentioned verbatim.**
- **Compare the EBI name mentioned with the grid of Evidence-Based Interventions. If unsure about the correct title, display the SHOW CARD and ask the Key Informant to confirm the title in the Grid of EBIs.**

|  | **Name of EBI mentioned by Key Informant** | **Name of EBI from grid, if available.** |
| --- | --- | --- |
| 1 |  |  |
| 2 |  |  |
| 3 |  |  |
| 4 |  |  |
| 5 |  |  |
| 6 |  |  |
| 7 |  |  |
| 8 |  |  |
| 9 |  |  |
| 10 |  |  |

| **EVIDENCE-BASED INTERVENTIONS** | | | |
| --- | --- | --- | --- |
| Mental Health Programs | | | |
| 1 | *Acceptance and Commitment Therapy (ACT)* | 20 | *Interpersonal Therapy (IPT) (offered as a stand-alone EBI)* |
| 2 | *Acceptance-Based Behavioral Therapy for Generalized Anxiety Disorder* | 21 | *Life Goals Collaborative Care (LGCC)* |
| 3 | *Applied Suicide Intervention Skills Training (ASIST)* | 22 | *Mental Health First Aid* |
| 4 | *Assertive Community Treatment (ACT) --- adapted to later become Full Service Partnerships* | 23 | *Mindfulness-Based Cognitive Therapy (MBCT)* |
| 5 | *Cognitive Behavioral Social Skills Training (CBSST)* | 24 | *Mindfulness-Based Stress Reduction (MBSR)* |
| 6 | *Cognitive Behavioral Therapy – (group format; stand-alone EBI, no particular title)* | 25 | *National Alliance on Mental Illness (NAMI) Family-to-Family Education Program* |
| 7 | *Cognitive Behavioral Therapy – (individual format; stand-alone EBI, no particular title)* | 26 | *PEARLS (Program to Encourage Active, Rewarding Lives for Seniors)* |
| 8 | *Cognitive Behavioral Therapy – Trauma Focused (TF-CBT)* | 27 | *Prevention of Suicide in Primary Care Elderly: Collaborative Trial (PROSPECT)* |
| 9 | *Cognitive Behavioral Therapy for Late-Life Depression* | 28 | *Problem Solving Treatment/Therapy (PST) (offered as a stand-alone EBI)* |
| 10 | *Computer-Assisted System for Patient Assessment and Referral (CASPAR)* | 29 | *Prolonged Exposure Therapy for Posttraumatic Stress Disorders* |
| 11 | *Computer-Based Cognitive Behavioral Therapy (Beating the Blues)* | 30 | *Psychiatric Rehabilitation Process Model* |
| 12 | *Crisis-Oriented Recovery Services (CORS)* | 31 | *Question, Persuade, and Refer (QPR) Gatekeeper Training for Suicide Prevention* |
| 13 | *Critical Time Intervention - Individual format (CBT)* | 32 | *Recognizing and Responding to Suicide Risk (RRSP)* |
| 14 | *Depression Prevention (Managing Your Mood)* | 33 | *Senior Reach* |
| 15 | *Eye Movement Desensitization and Reprocessing (EMDR)* | 34 | *Stress-Busting Program for Family Caregivers* |
| 16 | *Functional Adaptation Skills Training (FAST)* | 35 | *Suicide Prevention Training for Service Providers* |
| 17 | *Healthy IDEAS (Identifying Depression, Empowering Activities for Seniors)* | 36 | *Systems Training for Emotional Predictability and Problem Solving (STEPPS)* |
| 18 | *Improving Mood –Promoting Access to Collaborative Treatment (IMPACT)* | 37 | *Telemedicine-Based Collaborative Care* |
| 19 | *Integrated Illness Management and Recovery (I-IMR)* | 38 | *Wellness Recovery Action Plan (WRAP)* |
| Substance Use Programs, with and without Mental Health components | | | |
| 39 | *12-Step Facilitation Therapy* | 54 | *Motivational Enhancement Therapy (MET)* |
| 40 | *Alcohol Behavioral Couple Therapy (ABCT)* | 55 | *Network Support Treatment (NST) for Alcohol Dependence* |
| 41 | *Anger Management for Substance Abuse and Mental Health Clients: A CBT Manual* | 56 | *Pathways’ Housing First Program* |
| 42 | *Brief Strengths-Based Case Management for Substance Abuse* | 57 | *Prevention and Management of Alcohol Problems in Older Adults* |
| 43 | *Broad Spectrum Treatment (BST) and Naltrexone for Alcohol Dependence* | 58 | *Prize Incentives Contingency Management for Substance Abuse* |
| 44 | *Cocaine-Specific Coping Skills Training (CST)* | 59 | *Project ASSERT (Alcohol and Substance Abuse Services, Education, and Referral to Treatment)* |
| 45 | *Dialectical Behavior Therapy* | 60 | *Relapse Prevention Therapy (RPT)* |
| 46 | *Dynamic Deconstructive Psychotherapy* | 61 | *Screening, Brief Intervention, and Referral to Treatment Model (SBIRT )* |
| 47 | *Friends Care* | 62 | *Seeking Safety (SS)* |
| 48 | *GET SMART/UPBEAT* | 63 | *Service Outreach and Recovery (SOAR)* |
| 49 | *Interactive Journaling* | 64 | *Solution-Focused Group Therapy* |
| 50 | *Interim Methadone Maintenance* | 65 | *Supportive-Expressive Psychotherapy* |
| 51 | *Matrix Intensive Outpatient Program for the Treatment of Stimulant Abuse* | 66 | *TEAMcare* |
| 52 | *Methadone Maintenance Treatment (Narcotic Treatment Program)* | 67 | *Trauma-Informed Substance Abuse Treatment for Women (Boston Consortium Model)* |
| 53 | *Motivational Interviewing (MI)* | 68 | *Woman's Path to Recovery* |
| **EVIDENCE-BASED INTERVENTIONS** | | | |
| Disease Prevention, Health Promotion and Chronic Disease Management | | | |
| 69 | *A Matter of Balance (MOB): Managing concerns about falls* | 91 | *HomeMeds* |
| 70 | *Active Choices* | 92 | *Medication Management Improvement System (MMIS)* |
| 71 | *Active Living Everyday (ALED)* | 93 | *MedOptz* |
| 72 | *Arthritis Foundation Aquatic Program* | 94 | *National Diabetes Prevention Program (NDPP)* |
| 73 | *Arthritis Foundation Exercise Program* | 95 | *New York University Caregiver Intervention (NYUCI)* |
| 74 | *Arthritis Foundation Tai Chi Program* | 96 | *Otago Exercise Programme* |
| 75 | *Arthritis Self-Management (Self-Help) Progam (ASMP)* | 97 | *Positive Self-Management for HIV (PSMP)* |
| 76 | *Brief Intervention & Treatment for Elders (BRITE)* | 98 | *Powerful Tools for Caregivers* |
| 77 | *Care Transitions Intervention (CTI)* | 99 | *PROFET (Prevention of Falls in the Elderly Trial)* |
| 78 | *Cuidando con Respeto (Caring with Respect)* | 100 | *Program of All-Inclusive Care for the Elderly (PACE)* |
| 79 | *Disease Prevention and Health Promotion Program (DPHP)* | 101 | *Resources for Enhancing Alzheimer's Caregiver Health II (Reach II)* |
| 80 | *EnhanceFitness* | 102 | *SAFE Health Behavior and Exercise Intervention* |
| 81 | *EnhanceWellness* | 103 | *Savvy Caregiver* |
| 82 | *Erlangan Fitness Intervention* | 104 | *Stay Active and Independent for Life (SAIL)* |
| 83 | *Falls Management Exercise (FaME) Intervention* | 105 | *Stay Safe, Stay Active* |
| 84 | *FallScape* | 106 | *Stepping On* |
| 85 | *A Matter of Balance (MOB): Managing concerns about falls* | 107 | *Tai Ji Quan: Moving for Better Balance* |
| 86 | *FallsTalk* | 108 | *Tai-Chi: Moving for Better Balance* |
| 87 | *Fit and Strong!* | 109 | *TCARE® Support System (Tailored Caregiver Assessment & Referral®)* |
| 88 | *Geri-Fit® Strength Training Workout* | 110 | *Veterans Affairs Group Exercise Program* |
| 89 | *Healthier Living: Managing Ongoing Health Conditions* | 111 | *Walk with Ease (group program and self-directed program)* |
| 90 | *Healthy Moves for Aging Well* |  |  |
| Stanford Chronic Disease Self-Management Programs (CDSMPs) | | | |
| 112 | *Chronic Disease Self-Management Program (CDSMP)* | 116 | *Diabetes Self-Management Program (DSMP)* |
| 113 | *Chronic Pain Self-Management Program (CPSMP)* | 117 | *Tomando Control de su Salud (Spanish Chronic Disease Self-Management Program)* |
| 114 | *Better Choices, Better Health - Arthritis (online Arthritis Self-Management Program)* | 118 | *Programa de Manejo Personal de la Diabetes (Spanish Diabetes Self-Management Program)* |
| 115 | *Better Choices, Better Health - Chronic Disease (online CDSMP)* | 119 | *Programa de Manejo Personal de la Artritis (Spanish Arthritis Self-Management Program)* |

Q7 Now that you have identified the EBIs, we’d like to understand how organizations come to know that these are evidence-based interventions compared to other practices that you do not consider evidence-based.

In other words, how does your organization come to identify these interventions as evidenced-based? **(Probe for specific indicators or impressions on how the respondent arrives at the definition of *evidence-based practice* or *evidence-based intervention*.)**

**Probes:**

- **A person and/or entity has identified the interventions as evidence-based (e.g., a specific researcher or research organization, a national or international clearinghouse or registry of EBIs, a funding agency or reimbursement entity, etc.);**
- **The process by which these interventions are identified and selected as evidence-based (e.g., rigorous evaluation using standardized methods, results are published in an academic journal, a manual or treatment guide is available, etc.);**
- **Any other definition or impression the respondent may have of how the agency/organization comes to identify these interventions as “evidence-based practice” or “evidence-based intervention.” Also probe for any terms the respondent uses such as “evidence-informed,” “community-informed,” “promising,” etc.**

|  |
| --- |

**SECTION 2: QUESTIONS ABOUT EACH EBI.**

I will ask the next set of questions about each EBI that you mentioned, starting with the first one: [NAME OF EBI]

Q8 What year did your organization start [EBI NAME]? /__________/ (yyyy)

Q9 Where is it held? (What setting?) **Display the SHOW CARD, and check all that apply.**

|  | Hospital |
| --- | --- |
|  | Clinic |
|  | Adult Day Healthcare Centers / PACE sites |
|  | Social or human service organization |
|  | Congregate meal site |
|  | Senior center or community center |
|  | Recreational facility (i.e., YMCA, city or county park) |
|  | Private residence |
|  | Public housing units (e.g., subsidized housing) |
|  | Board-and-care facilities |
|  | Senior independent living/retirement facility |
|  | Nursing home / Assisted living facility |
|  | Place of worship (i.e., church, synagogue, temple, etc.) |
|  | Provided remotely using tele-technology (phone, videoconferencing, etc.) |
|  | Other *–specify:* |
|  | Other *–specify:* |

Q10 Specify the location(s) where this EBI is offered. In other words, where would I direct someone who needs to use this EBI? **[If respondent cannot provide name and address, then ask for closest intersection. Use back of paper if additional space is needed to list additional locations.]**

| Name and Address of Location 1 where EBI is offered: |
| --- |
| Name and Address of Location 2 where EBI is offered: |
| Name and Address of Location 3 where EBI is offered: |
| Name and Address of Location 4 where EBI is offered: |
| Name and Address of Location 5 where EBI is offered: |
| Name and Address of Location 6 where EBI is offered: |
| Name and Address of Location 7 where EBI is offered: |

Q11 How many people does this program serve per year?

/_______/ (Enter number; use most recent year if the amount varies)

Q12 Has the number of [EBI NAME] participants in a year increased, decreased, or stayed about the same over the past three years?

|  | Increased |
| --- | --- |
|  | Decreased |
|  | Stayed about the same |
|  | Program was just implemented, too soon to tell |
|  | Other/Comment – specify: |

Q13 What populations (or groups) is this EBI specifically targeting or intended for? **[Probe for criteria such as: age, language, race/ethnicity, income, specific disease/condition, living situation (i.e., private residence vs. nursing home), etc.]**

|  |
| --- |

Q14 This question focuses on the utilization of this EBI, by specific demographics. Let us know if your organization does not collect any of this specific information. If you do not know the specific proportions of participants, please let us know. We can follow up with you for the specific information, if it is not readily available during the interview. Of the participants in [EBI name], indicate the proportion that are . . . ? **[Display the SHOW CARD; Check all that apply, and indicate the %, NC for Not Collected, or DK for Don’t Know.]**

|  | **Specific demographics** | **%** |
| --- | --- | --- |
|  | Adults ages 18 – 49 years |  |
|  | Adults ages 50 – 59 years |  |
|  | Adults ages 60 and older |  |
|  |  |  |
|  | Gender (female) |  |
|  |  |  |
|  | Non-Hispanic White |  |
|  | Hispanic/Latino |  |
|  | African-American |  |
|  | Asian/Pacific Islander |  |
|  | Native American/American Indian |  |
|  | Mixed race or other |  |
|  |  |  |
|  | Primary language is English |  |
|  | Primary language is Spanish |  |
|  | Primary language is Vietnamese |  |
|  | Primary language is Other Language |  |
|  |  |  |
|  | Non-insured (e.g., do not have any type of health insurance) |  |
|  |  |  |
|  | U.S. Citizen |  |
|  | Permanent Resident/Green Card |  |
|  | Refugee/Asylum |  |
|  | Residency application is in process (awaiting legal residence) |  |
|  | Visa (B1/B2, A3, G5, J1, Tourist, U-Visa, T-Visa, Other Visa) |  |
|  | A border crossing card |  |
|  | Undocumented |  |
|  | TPS (Temporary Protected Status) |  |

Q15 Please describe the geographic reach or catchment area of [EBI name]. Be as specific as possible in describing the service catchment area by indicating the health district, city, and /or zip code within each of the following four Service Planning Areas (SPAs): 4, 6, 7, and 8.

**Display the map of County SPAs and Health Districts, and mark all that apply. Note: The service catchment area and location where the intervention is offered are separate questions.**

| ***Cities & Communities Within SPA 4 (Metro), per Health District*** | | | | |
| --- | --- | --- | --- | --- |
| ***Health District:*** | ***Central*** | ***Hollywood-Wilshire*** | ***Northeast*** |  |
|  | *Los Feliz* | *Hancock Park* | *Boyle Heights* |  |
|  | *Korea Town* | *Hollywood Hills* | *Eagle Rock* |  |
|  | *Chinatown* | *Hollywood* | *El Sereno* |  |
|  | *Echo Park* | *Park La Brea* | *Glassell Park* |  |
|  | *Westlake* | *Mount Olympus* | *Highland Park* |  |
|  | *Silverlake* |  | *Monterey Hills* |  |
| ***Cities & Communities Within SPA 7 (East), per Health District*** | | | | |
| ***Health District:*** | ***Bellflower*** | ***East L.A.*** | ***San Antonio*** | ***Whittier*** |
|  | *Artesia* | *City of Commerce* | *Bell* | *La Habra Heights* |
|  | *Bellflower* | *East Los Angeles* | *Bell Gardens* | *Pico Rivera* |
|  | *Cerritos* | *Montebello* | *Cudahy* | *Santa Fe Springs* |
|  | *Hawaiian Gardens* |  | *Downey* |  |
|  | *Lakewood* |  | *Huntington Park* |  |
|  | *Norwalk* |  | *Maywood* |  |
|  | *Signal Hill* |  | *South Gate* |  |
|  |  |  | *Vernon* |  |
|  |  |  | *Walnut Park* |  |
| ***Health Districts, Cities & Communities Within SPA 6 (South)*** | | | | |
| ***Health District:*** | ***Southwest*** | ***Southeast*** | ***South*** | ***Compton*** |
|  | *Crenshaw* | *Florence* | *Florence-Graham* | *Compton* |
|  | *University* | *Jefferson* | *Watts* | *Paramount* |
|  | *Baldwin Hills* |  | *Firestone Park* | *Willowbrook* |
|  | *Leimert Park* |  |  | *Lynwood* |
|  | *View Park-*  *Windsor Hills* |  |  |  |
|  | *Village Green* |  |  |  |
|  | *Cienega* |  |  |  |
|  | *St. James Park* |  |  |  |
| ***Health Districts, Cities & Communities Within SPA 8 (South Bay)*** | | | | |
| ***Health District:*** | ***Inglewood*** | ***Torrance*** | ***Harbor*** | ***Long Beach*** |
|  | *Inglewood* | *Manhattan Beach* | *Palos Verdes* | *North Long Beach* |
|  | *Lennox* | *Hermosa Beach* | *Rolling Hills* | *Bixby Knolls* |
|  | *Hawthorne* | *Redondo Beach* | *Rancho Palos Verdes* | *Long Beach* |
|  | *El Segundo* | *Torrance* | *San Pedro* | *San Pedro* |
|  | *Gardena* | *Lomita* | *Wilmington* | *California Heights* |
|  | *Westmont* | *Palos Verdes Estates* |  | *Los Altos* |
|  | *Athens* | *Harbor City* |  |  |
|  |  | *Carson* |  |  |
|  |  | *Rancho Dominguez* |  |  |
|  |  | *Keystone* |  |  |

Q16 Does your EBI require that providers/interventionists follow a manual or intervention/treatment guide? If so, briefly describe.

|  | **Is Manual/guide required?** | **Describe:** |
| --- | --- | --- |
|  | No | N/A |
|  | Yes |  |

Q17 What level of training is required to deliver this program (at the client/participant level)? **Display the SHOW CARD, and check all that apply.**

|  | *No specialized training required* |
| --- | --- |
|  | *Brief training / orientation session* |
|  | *Certification from program developers* |
|  | *Previous clinical experience* |
|  | *Education or degree requirement (i.e., must have a master’s degree, RN, or other degree)* |
|  | *Other training – specify:* |

**[INTERVIEWER: Now go back and ask questions # 9 – 17 again, but for the next EBI on the list.]**

**SECTION 3. Evaluation and Implementation Issues.**

Q18 In general, describe how your organization evaluates these EBIs. You may select one EBI as an example, or describe the evaluation of EBIs in general. **[First document what they say, without reading the probes. Then read the probes and document the responses in the space provided. Use additional paper if needed.]**

**_________________________________________________________________________________________________________________________________________________________________________________________________________________________________________________________________________________________________________________________________________________________________________________________________**

|  | ***Probes:*** | ***Document their responses here:*** |
| --- | --- | --- |
|  | What specific client outcomes are measured?  **(Probe for specific data collection items, scales, instruments, or forms)** |  |
|  | What specific organizational outcomes are measured?  **(Probe for specific data collection items, scales, instruments, or forms)** |  |
|  | How does your organization/staff utilize outcomes data?  (e.g., to track client treatment plans/goals, to track expenses/claims for funding purposes, etc.) |  |
|  | Does evaluation for this program require any electronic management information system? If so, describe. |  |

Q19 What additional information would be helpful to evaluate EBIs? If you could have any information that would help you determine how effective a program is, what information would that be? How would this information be helpful to you? Why is this information important to your organization?

|  | **List any additional information that would be helpful to evaluate EBIs** | **List reasons why this information is important to the respondent, clinicians, and/or the organization** |
| --- | --- | --- |
|  |  |  |
|  |  |  |
|  |  |  |
|  |  |  |
|  |  |  |

Q20 Did your organization modify, adapt, or specifically tailor any of the EBIs from the original format? If so, in what ways were programs modified? **[First document what they say, without reading the probes. Then display the SHOW CARD, read the probes, and check all that apply. Use additional paper if needed.]**

**___________________________________________________________________________________________________________________________________________________________________________________________________________________________________________________________________________________________________________________________________________________________________________________________________________________________________________________________________________________________________________________________________________________________**

|  | No adaptation or modification was made to the original EBPs |
| --- | --- |
|  | Modified to address values and/or issues relevant to aging or the aging process |
|  | Offered in a different language or in a bilingual format |
|  | Modified to the needs of persons with low literacy and numeracy |
|  | Modified to address cultural values and/or issues related to the lived experience of specific social groups in your community (i.e., ethnic/racial minorities, homeless persons, LGBTQ) (e.g., modified the role of the facilitator to be attuned to cultural values; incorporated culturally-specific communication styles within sessions) |
|  | Length: Changed the number of sessions, class meetings, or time spent in any given session or meeting (e.g., added another session at the end; decreased the total number of sessions or encounters suggested by the developers; 2 hour sessions rather than 3 hour sessions, etc.) |
|  | Offered in conjunction with another program (e.g., in conjunction with case management services, adult day health care, medication management, etc.) |
|  | Trained non-traditional personnel to offer the program (utilized personnel not initially intended to provide the program as first indicated by the program developers) |
|  | Other modifications *– specify:* |
|  | Other modifications *– specify:* |

Q21 How are EBIs in your organization financially supported? You can indicate more than one source of funding. **Display the SHOW CARD, and check all that apply.**

|  | *Medicare* |
| --- | --- |
|  | *Medi-Cal (Medicaid)* |
|  | *Private insurance* |
|  | *Participant payments (fee-for-service, co-payments, private pay, etc.)* |
|  | *County, state, or federal grant programs* |
|  | *Foundation or corporation funding* |
|  | *Other – specify:* |
|  | *Other – specify:* |

Q22 Describe typical organizational costs related to the implementation of EBIs for older adults.

**[First document what they say, without reading the probes. Then read the probes and document the responses in the space provided. Use additional paper if needed.]**

**________________________________________________________________________________________________________________________________________________________________________________________________________________________________________________________________________________________________________________________________________________________________________________________________________________________________________________________________________________________________________________________________________________________________________________________________________________________________________**

|  | ***Probes:*** | ***Document their responses here:*** |
| --- | --- | --- |
|  | FTEs, training manuals, training hours, transportation/mileage, other costs? |  |
|  | For your organization, what have been the most significant costs in implementing EBIs for older adults? |  |
|  | Describe any unexpected costs your organization had to deal with to implementing any particular EBI for older adults. |  |

Q23 What factors have ultimately influenced or facilitated your organization’s decision or ability to deliver EBIs for older adults? **[First document what they say, without reading the probes. Then display the SHOW CARD, read the probes, and check all that apply. Use additional paper if needed.]**

**_____________________________________________________________________________________________________________________________________________________________________________________________________________________________________________________________________________________________________________________________________________________________________________________________________________________________________________________________________________________________________________________________________________________________________________________________________________________________________________________________________________________________________________________**

|  | Resources / Funding (e.g., funders or grantors identified these EBIs as a requirement for providing services; funding was made available to deliver the EBI, such as a contract bid, funding application, reimbursement, etc.) |
| --- | --- |
|  | Organizational support (e.g., organizational champion for the EBI; strong leadership, etc.) |
|  | Organizational partnerships, collaborations, and/or influences (e.g., collaborated with a researcher to offer intervention to our clients/consumers; partnered with other agency/organization to offer the intervention or to offset operational costs) |
|  | Nature of the EBIs (e.g., how the EBI works appeared theoretically sound; the research behind the EBI was adequate; acceptable to our target population(s)) |
|  | Client/consumer preferences (e.g., the EBI was closely allied to our client/consumer norms, needs, preferences, and characteristics; potential for moderate to high acceptance of target population) |
|  | Other facilitating factors – specify: |
|  | Other facilitating factors – specify: |

Q24 Describe the challenges or barriers that your agency/organization has faced in implementing EBIs for older adults. **[First document what they say, without reading the probes. Then read the probes, and check all that apply. Use additional paper if needed.]**

**_____________________________________________________________________________________________________________________________________________________________________________________________________________________________________________________________________________________________________________________________________________________________________________________________________________________________________________________________________________________________________________________________________________________________________________________________________________________________________________________________________________________________________________________**

|  | ***Probes:*** | ***Document their responses here:*** |
| --- | --- | --- |
|  | Lack of resources (funding, staff, etc.) |  |
|  | Lack of infrastructure within the organization (systems, processes, etc.) |  |
|  | Staff resistance; Lack of leadership |  |
|  | Challenges/problems with organizational partners/collaborators |  |
|  | Relevance to our clients; difficulty implementing or adapting EBI to our clients |  |
|  | Other challenges/barriers – Specify: |  |
|  | Other challenges/barriers – Specify: |  |

Q25 Are there unique issues to consider in the implementation of EBIs for *older* adults in comparison to EBIs for adults in general (e.g., any unique challenges/barriers, costs, opportunities/facilitators)?

|  |
| --- |

Q26 Are there unique issues to consider in the implementation of EBIs for *minority* older adults in comparison to non-minority older adults (e.g., any unique challenges/barriers, costs, opportunities/facilitators)?

|  |
| --- |

**SECTION 4. PAST AND FUTURE EBIs.**

Q27 What EBIs were offered in the past 5 years, but are now discontinued?

Why were they discontinued?

|  | **Name of EBI that was discontinued** | **Reason(s) for discontinuing** |
| --- | --- | --- |
|  |  |  |
|  |  |  |
|  |  |  |
|  |  |  |
|  |  |  |

Q28 What would it take to adopt an additional EBI for older adults in your agency/organization?

|  | Which EBI would that be? | What do you anticipate may help to implement this additional EBI? | What do you anticipate may hinder to implement this additional EBI? |
| --- | --- | --- | --- |
|  |  |  |  |
|  |  |  |  |
|  |  |  |  |
|  |  |  |  |

Q29 Is there anything else about the adoption of EBIs for older adults that you think would be important for us to know.

|  |
| --- |

Q30 Is there anything else you'd like to share about your personal experience in the design or implementation of any of the programs we have talked about?

|  |
| --- |

**END OF INTERVIEW.** Thank you for participating in this interview. We appreciate your time and insights.
